# Sphingomyelinase‐Mediated Multitimescale Clustering of Ganglioside GM1 in Heterogeneous Lipid Membranes

**DOI:** 10.1002/advs.202101766

**Published:** 2021-09-02

**Authors:** Hyun‐Ro Lee, Siyoung Q. Choi

**Affiliations:** ^1^ Department of Chemical and Biomolecular Engineering Korea Advanced Institute of Science and Technology (KAIST) Daejeon 34141 Republic of Korea; ^2^ KAIST Institute for the NanoCentury Korea Advanced Institute of Science and Technology (KAIST) Daejeon 34141 Republic of Korea

**Keywords:** ceramides, GM1 gangliosides, lipid clustering, lipid membranes, membrane remodeling, sphingomyelinases

## Abstract

Several signaling processes in the plasma membrane are intensified by ceramides that are formed by sphingomyelinase‐mediated hydrolysis of sphingomyelin. These ceramides trigger clustering of signaling‐related biomolecules, but how they concentrate such biomolecules remains unclear. Here, the spatiotemporal localization of ganglioside GM1, a glycolipid receptor involved in signaling, during sphingomyelinase‐mediated hydrolysis is described. Real‐time visualization of the dynamic remodeling of the heterogeneous lipid membrane that occurs due to sphingomyelinase action is used to examine GM1 clustering, and unexpectedly, it is found that it is more complex than previously thought. Specifically, lipid membranes generate two distinct types of condensed GM1: 1) rapidly formed but short‐lived GM1 clusters that are formed in ceramide‐rich domains nucleated from the liquid‐disordered phase; and 2) late‐onset yet long‐lasting, high‐density GM1 clusters that are formed in the liquid‐ordered phase. These findings suggest that multiple pathways exist in a plasma membrane to synergistically facilitate the rapid amplification and persistence of signals.

## Graphical abstract

This work investigates how sphingomyelinase‐mediated hydrolysis of sphingomyelin concentrates ganglioside GM1 in heterogeneous lipid membranes. The sphingomyelinase reaction aggregates GM1 at different spatiotemporal scales via two mechanisms: 1) early, temporary GM1 clustering in ceramide‐rich domains; and 2) late, permanent GM1 clustering in cholesterol‐rich lipid rafts. These multiple clustering pathways can synergistically facilitate the amplification and persistence of signals.

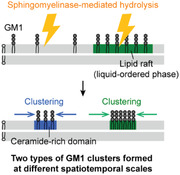

## 1 Introduction

Ceramides are essential membrane lipids that regulate diverse cell‐signaling events. In response to various receptor‐mediated or external stimuli,^[^ ^1^, ^2^, ^3^, ^4^, ^5^, ^6^ ^]^ ceramides are generated in the outer leaflet of the plasma membrane through hydrolysis of sphingomyelin (SM) by sphingomyelinases (SMases). Accumulated ceramides are known to modify membrane biophysical properties^[^ ^7^, ^8^, ^9^ ^]^ and to coalesce signaling‐related biomolecules (e.g., membrane receptors,^[^ ^2^, ^10^, ^11^, ^12^ ^]^ ion channels,^[^ ^13^, ^14^ ^]^ caveolin‐1,^[^ ^15^ ^]^ and gangliosides^[^ ^3^, ^16^, ^17^ ^]^) into specialized microdomains. These ceramide‐rich microdomains are gel phases characterized by high shear viscosity, which effectively slow the lateral diffusion of the captured signaling biomolecules.^[^ ^18^ ^]^ This ceramide‐induced clustering is thought to amplify signaling events,^[^ ^19^, ^20^, ^21^ ^]^ thus eliciting physiological and pathological responses such as apoptosis,^[^ ^22^, ^23^, ^24^ ^]^ T‐cell immune responses,^[^ ^25^, ^26^ ^]^ neurodegeneration,^[^ ^16^, ^27^, ^28^ ^]^ and endothelial dysfunction.^[^ ^29^ ^]^

Despite the importance of ceramides in cell signaling, the fundamental mechanism of ceramide‐associated coalescence of signaling biomolecules remains unknown. Previous studies observed that specific signaling biomolecules cluster into ceramide‐rich microdomains at particular times or at equilibrium.^[^ ^3^, ^17^, ^30^, ^31^ ^]^ However, the series of processes whereby signaling biomolecules are localized into ceramide‐rich microdomains is undefined. The signaling cascades activated by the clustering of signaling biomolecules can be affected by the location and size of these microdomains, in addition to the areal density and dwelling time of the signaling biomolecules in the microdomains. It is therefore essential to scrutinize the spatiotemporal distribution of signaling biomolecules in the plasma membrane. Several previous studies have suggested that SMase‐mediated hydrolysis of SM provokes the liquid‐to‐gel phase transition of highly ordered lipid domains or lipid rafts, which is followed by their aggregation into large ceramide‐rich microdomains.^[^ ^32^, ^33^, ^34^, ^35^ ^]^ This aggregation is thought to be responsible for the clustering of signaling biomolecules, but there is insufficient experimental evidence. Furthermore, because SMase activity depends on membrane phase and composition,^[^ ^36^, ^37^ ^]^ the distinct condensation kinetics of signaling biomolecules throughout heterogeneous membranes, which are separated into different regions, must be identified.

To this end, we demonstrate the spatiotemporal localization of ganglioside GM1 in response to SMase activation. GM1 is a glycosphingolipid receptor that modulates various cellular functions, including pathogenic endocytosis,^[^ ^38^, ^39^ ^]^ neurotrophic action,^[^ ^40^, ^41^ ^]^ growth‐factor signaling,^[^ ^42^ ^]^ and integrin signaling.^[^ ^43^ ^]^ GM1 is found mainly in lipid rafts present in the outer plasma membrane leaflet, thus commonly used as a marker of the lipid rafts that contain various signaling proteins.^[^ ^3^, ^44^, ^45^ ^]^ Using an ultrastable freestanding lipid membrane array,^[^ ^46^ ^]^ we visualized overall dynamic GM1 clustering during SMase hydrolysis in real time. We found that SMase activity concentrates GM1 in the outer leaflet within lipid membranes via the following two mechanisms: 1) early, temporary GM1 clustering in the ceramide‐rich domains nucleated from the liquid‐disordered (L_d_) phase (nonraft‐like regions); and 2) late, permanent GM1 condensation in the liquid‐ordered (L_o_) phase (raft‐like regions). Our findings suggest that the GM1 clustering/condensation in heterogeneous lipid membranes occurs at multiple spatiotemporal scales. SMase‐mediated ceramide generation thus controls the duration and surface density of the spatial association of signaling molecules, depending on the heterogeneous distribution of lipids in the plasma membrane.

## 2 Results

### 2.1 A Lipid Membrane Platform to Visualize GM1 Clustering Dynamics

**Figure 1:**
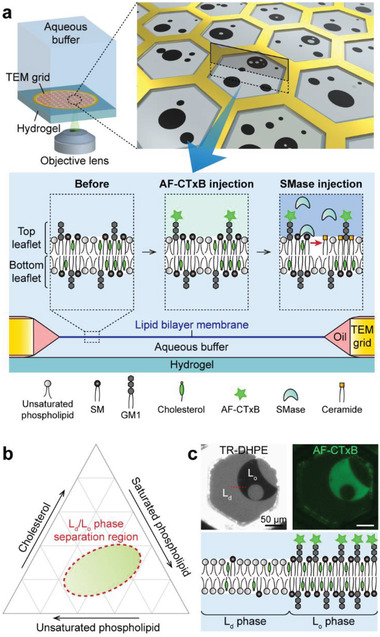
Scheme of our freestanding, planar lipid membrane system for the analysis of sphingomyelinase (SMase)‐mediated membrane remodeling. a) Tens of lipid membranes were created in the hexagonal holes of the transmission electron microscopy (TEM) grid. As the aqueous buffer below the lipid membranes was isolated from that above the lipid membranes, Alexa Fluor 488‐labeled cholera toxin subunit B (AF–CTxB) injected into the aqueous buffer above the lipid membranes combined only with ganglioside GM1 present in the top membrane leaflet. Similarly, SMase acted only on the top membrane leaflet. SMase‐induced GM1 redistribution was observed through inverted fluorescence microscopy in real time. Detailed experimental processes are introduced in Figure S1 (Supporting Information). b) The lipid mixtures approximately located in the dotted‐line region of the phase diagram are segregated into liquid‐disordered (L_d_) and liquid‐ordered (L_o_) phases at equilibrium. c) Representative fluorescence images of the lipid membranes before the SMase reaction. Two liquid phases are easily differentiated by Texas Red–1,2‐dihexadecanoyl‐*sn*‐glycero‐3‐phosphoethanolamine (TR–DHPE) and AF–CTxB fluorescence.

We created an ultrastable freestanding planar lipid membrane array in a transmission electron microscopy (TEM) grid (**Figure** **1**a and Figure S1 (Supporting Information)).^[^ ^46^ ^]^ The prepared lipid membranes consisted of GM1, a mixture of phospholipids of biological relevance, cholesterol, and fluorescently labeled lipids. The large, freestanding, flat membrane structure enabled us to visualize overall membrane dynamics through fluorescence microscopy in real time. GM1 specifically bound Alexa Fluor 488‐labeled cholera toxin subunit B (AF–CTxB) by hydrogen bonding between the pentasaccharide moiety of GM1 and B‐pentamer of CTxB,^[^ ^47^, ^48^, ^49^ ^]^ and thus we could monitor GM1 distribution by detecting AF–CTxB. Because GM1 is predominantly located in the outer leaflet of the plasma membrane, we utilized the unique feature of our lipid membrane system to specifically visualize the GM1 present in the top leaflet of the lipid membranes. As shown in the bottom of Figure 1a, the aqueous buffer below the lipid membrane is surrounded by the lipid membrane, oil, the TEM grid, and hydrogel, so it is isolated from the aqueous buffer above the lipid membrane. Therefore, AF–CTxB injected into the aqueous buffer above the lipid membranes cannot diffuse below the lipid membranes, and thus binds only to GM1 that is present in the top membrane leaflet. This affords exclusive detection of the GM1 distribution in the top membrane leaflet.

We used methyl‐*β*‐cyclodextrin, a cholesterol transporter, to fix the cholesterol concentration in the lipid membranes at ≈30 mol%, as cholesterol comprises 20–50 mol% of the total lipids in mammalian cell membranes.^[^ ^50^, ^51^ ^]^ At this cholesterol concentration and at 23 °C, L_d_ and L_o_ phases coexisted within the lipid membranes, unless the molar ratio of unsaturated and saturated phospholipids was exceptionally high or low (the dotted‐line region shown in Figure 1b).^[^ ^52^ ^]^ The L_d_ phase was enriched in unsaturated phospholipids and fluorescently labeled lipids, such as Texas Red–1,2‐dihexadecanoyl‐*sn*‐glycero‐3‐phosphoethanolamine (TR–DHPE), and the L_o_ phase was enriched in saturated phospholipids, cholesterol, and GM1.

Due to variation in the partitioning of lipids into the two liquid phases, these phases were easily differentiated by fluorescence imagery. For example, because TR–DHPE was predominantly found in the L_d_ phase, the bright and dark membrane domains shown in the TR–DHPE image represent the L_d_ and L_o_ phases, respectively (Figure 1c). By contrast, GM1 was predominantly found in the L_o_ phase, such that the green fluorescence visible in the AF–CTxB image represents the L_o_ phase. Both domains should be transversely symmetric at equilibrium (bottom of Figure 1c), as this symmetric structure is thermodynamically favorable for such micrometer‐scale domains.^[^ ^53^ ^]^

### 2.2 SMase‐Mediated Reorganization of Lipid Membranes

**Figure 2:**
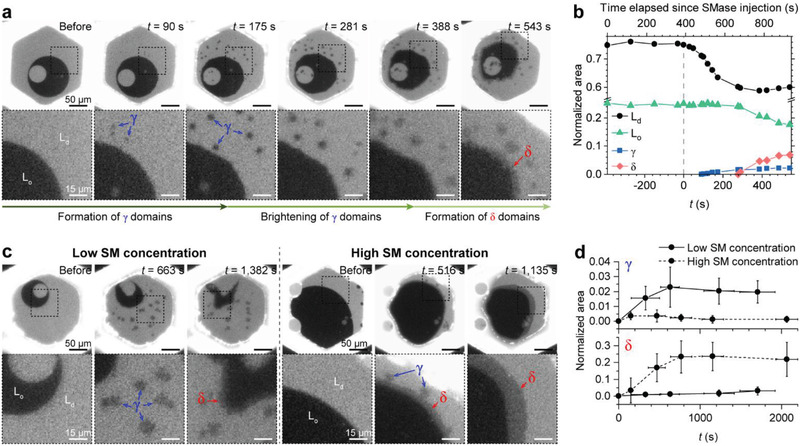
SMase‐induced dynamic remodeling of lipid membranes. a) Time‐lapse TR–DHPE fluorescence images of membrane reconstitution during SMase‐mediated hydrolysis. *γ* (blue arrow) and *δ* domains (red arrow) formed in sequence. The time (*t*) shown in the upper right of the images indicates the time elapsed since SMase began to remodel the lipid membranes. The images in the second row are enlarged views of the area surrounded by the black dotted square in the first row. b) The variation in the areas of the L_d_ and L_o_ phases and the *γ* and *δ* domains shown in (a) was measured over time. The areas were normalized to the initial lipid membrane area. c) The domain size varies with the molar fraction of sphingomyelin (SM). The representative lipid membranes containing low proportions of SM and high proportions of SM are shown in the left and right panels, respectively. d) The variation in the areas of the *γ* and *δ* domains of the lipid membranes containing low proportions of SM and high proportions of SM was measured over time. The areas were normalized to the initial lipid membrane area. The values are expressed as means ± standard deviations (the number of samples = 4–7 for *γ* and 3–7 for *δ*). The oil used to make the lipid membranes comprised dioleoyl phosphatidylcholine/dioleoyl phosphatidylserine/SM/dipalmitoyl phosphatidylserine/GM1 + TR–DHPE (60/10/25/4/1 + 1 mol% for (a), 65/10/20/4/1 + 1 mol% for lipid membranes containing low concentrations of SM (left panel of (c)), and 55/10/30/4/1 + 1 mol% for lipid membranes containing high concentrations of SM (right panel of (c)). Prior to the SMase‐mediated reaction, cholesterol was added to the lipid membranes via methyl‐*β*‐cyclodextrin.

In this system, SMase should only be able to hydrolyze SM in the top leaflet of the lipid membranes, because SMase cannot approach the bottom leaflet (bottom of Figure 1a). We intentionally designed this system to mimic the SMase‐mediated SM hydrolysis that occurs in the outer leaflet of the plasma membrane. At ≈400 s after the injection of a SMase solution, the L_d_ phase area decreased by around 12%, whereas the L_o_ phase area was largely unaffected (**Figure** **2**a,b and Video S1 (Supporting Information)). This reduction in the L_d_ phase area was presumably due to the decrease in area per molecule that occurred when SM was hydrolyzed to release ceramide^[^ ^54^, ^55^ ^]^ and to the condensation effect of ceramide.^[^ ^56^ ^]^ We denote the time when the membrane area begins to decrease as *t*, which is the time elapsed since SMase‐mediated hydrolysis of SM began (bottom axis in Figure 2b).

The SMase reaction also significantly changed the membrane organization, resulting in the formation of two new domains. First, at *t* ≈100 s, tiny dark domains heterogeneously nucleated in the L_d_ phase and grew to several micrometers in size (Figure 2a). We denote these *γ* domains, and they stopped growing at *t* ≈400 s. Intriguingly, most of the *γ* domains were initially as dark as the L_o_ phase, but gradually brightened into a gray color, which largely persisted. The brightened *γ* domains are distinct from the L_d_ and L_o_ phases, and have TR–DHPE fluorescence intensities that are between that of the L_d_ and L_o_ phases. Second, during the growth of *γ* domains in the L_d_ phase, the interface of the L_d_ and L_o_ phases became roughened. At *t* ≈300 s, gray regions that we denote *δ* domains were newly formed at the L_d_–L_o_ interface. Similar to the brighten *γ* domains, the *δ* domains also have fluorescence intensities that are between that of the L_d_ and L_o_ phases. The *δ* domains slowly grew toward the center of the L_o_ phase, reducing the L_o_ phase area. The growth rate of the *δ* domains markedly differed along the L_d_–L_o_ interface perimeter, which caused the irregular shape of the L_o_ phase.

We found that SMases sequentially created *γ* and *δ* domains in the various SM‐containing lipid membranes, which show that their creation was independent of the lipid composition of membranes (Figure S2, Supporting Information). However, the kinetics of domain formation depended on their lipid composition, particularly their concentration of SM. At a low SM concentration (left panel of Figure 2c), the area fraction of the L_d_ phase is relatively large, more *γ* domains nucleate in the L_d_ phase, and these grow to ≈10 µm in diameter. By contrast, at a high SM concentration (right panel of Figure 2c), the area fraction of the L_d_ phase is relatively small, and fewer *γ* domains nucleate in the L_d_ phase, and grow less. As shown in Figure 2d, the total area of the *γ* domains at a low SM concentration is an average of ≈4 times larger than that at a high SM concentration. In addition, at a lower SM concentration, the formation kinetics of *δ* domains are slow at the L_o_–L_d_ interface. By contrast, at high SM concentrations, the growth of the *δ* domains accelerates, and they are ≈10 times larger than at lower SM concentrations during the approximately same time, as shown in Figure 2c,d.

As shown in Figure S3 (Supporting Information), when the areas of the *γ* and *δ* domains are normalized to the areas of the L_d_ and L_o_ phases, respectively, the differences in the areas of the *γ* and *δ* domains are significantly reduced. In particular, the normalized areas of the *γ* and *δ* phases increase at a similar rate and to a similar extent over 500 s, regardless of the SM concentration. These results indicate that the areas of the *γ* and *δ* domains are greatly dependent on the areas of the L_d_ and L_o_ phases.

### 2.3 SMase‐Induced GM1 Crowding

**Figure 3:**
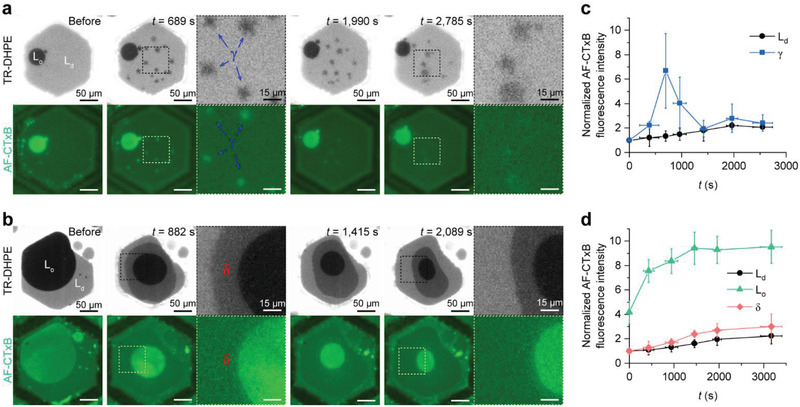
Dynamic clustering of ganglioside GM1 present in the top membrane leaflet during the SMase reaction. Time‐lapse fluorescence images of SMase‐induced GM1 redistribution in lipid membranes containing a) a low concentration of SM and b) a high concentration of SM. GM1 in the top membrane leaflet selectively bound AF–CTxB, and was thereby monitored by AF–CTxB fluorescence. The time (*t*) shown in the upper right of the images indicates the time elapsed since the SMase began to remodel the lipid membranes. c) The variation in the AF–CTxB fluorescence intensity of the L_d_ phase and the *γ* domain in the lipid membranes containing a low concentration of SM. d) The variation in the AF–CTxB fluorescence intensity of the L_d_ and L_o_ phases and the *δ* domain in the lipid membranes containing a high concentration of SM. The intensity values in (c) and (d) are normalized to that of the initial L_d_ phase and expressed as means ± standard deviations (the number of samples = 3–24 for (c) and 4–6 for (d)). The oil used to make the lipid membranes comprised dioleoyl phosphatidylcholine/dioleoyl phosphatidylserine/SM/dipalmitoyl phosphatidylserine/GM1 + Texas Red–1,2‐dihexadecanoyl‐*sn*‐glycero‐3‐phosphoethanolamine (65/10/20/4/1 + 1 mol% for membranes containing a low concentration of SM and 55/10/30/4/1 + 1 mol% for membranes containing a high concentration of SM). Prior to the SMase‐mediated reaction, cholesterol was added to the lipid membranes via methyl‐*β*‐cyclodextrin.

**Figure** **3** shows that during the reconstruction of the lipid membranes resulting from SMase‐mediated hydrolysis, GM1 present in the top membrane leaflet is redistributed. Because more *γ* domains are formed in the lipid membranes that contain a lower concentration of SM, as shown in the previous figures (Figure 2c,d), we examined the correlation of GM1 clustering with *γ* domains in these lipid membranes (Figure 3a). From Figure 3a, it can be seen that the AF–CTxB fluorescence intensity of the *γ* domains is greater than that of the L_d_ phase in the early stages of the SMase reaction, and then decreases as the reaction continues, whereas that of the L_d_ phase does not change considerably. Interestingly, Figure 3a also reveals that as the TR–DHPE fluorescence intensity of the *γ* domains increases (i.e., as they become gray in color), their AF–CTxB intensity decreases. Finally, after *t* ≈1500 s, the AF–CTxB fluorescence intensity of most of the *γ* domains is similar to that of the L_d_ phase, as shown in Figure 3a,c. These results indicate that GM1 that is present in the top membrane leaflet is initially condensed into *γ* domains, but that these are short‐lived.

The lipid membrane containing a high concentration of SM was used to visualize the GM1 clustering in large L_o_ phases and large *δ* domains, and the SMase‐induced GM1 redistribution in these areas over time is depicted in Figure 3b. Surprisingly, the AF–CTxB fluorescence intensity of the L_o_ phase increases to approximately twice its initial value as the area of the L_o_ phase decreases during SMase‐mediated hydrolysis of SM (Figure 3d). Assuming that the AF–CTxB fluorescence intensity is proportional to the areal density of GM1, this shows that the SMase reaction may result in approximately doubled concentrations of GM1 in the L_o_ phase in the top membrane leaflet. However, the area of the L_o_ phase decreases by more than 5 times (Figure S4, Supporting Information), whereas the AF–CTxB fluorescence intensity increases to less than 3 times its initial value. This implies that there might be a solubility limit to GM1 in the L_o_ phase, and that extremely dense GM1 might diffuse into the L_d_ phase and generate a temporary equilibrium. By contrast, the AF–CTxB fluorescence intensity of the *δ* domain remains approximately the same as that of the L_o_ phase throughout the reaction (Figure 3b,d), indicating that GM1 in the top membrane leaflet is not condensed into the *δ* domain. To understand why GM1 molecules in the top membrane leaflet cluster into only the *γ* domain and the L_o_ phase, we examined the phase behavior and structural variation of different regions in the following sections.

### 2.4 Phase Behavior of Separate Membrane Regions

**Figure 4:**
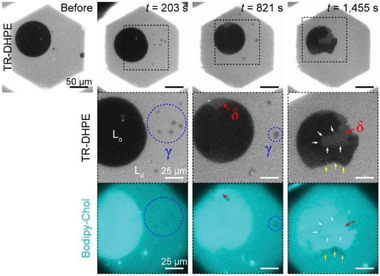
SMase‐induced cholesterol redistribution in lipid membranes. The lipid membrane undergoing SMase‐mediated hydrolysis was visualized by fluorescent labeling with TR–DHPE) and 23‐(dipyrrometheneboron difluoride)‐24‐norcholesterol (Bodipy‐Chol). The time‐lapse fluorescence images in the second and third rows are expanded views of the area outlined by the black dotted square in the first row. The time (*t*) indicates the time elapsed since SMase began to remodel the lipid membrane. The oil used to make the lipid membrane comprised dioleoyl phosphatidylcholine/dioleoyl phosphatidylserine/sphingomyelin/dipalmitoyl phosphatidylserine/ganglioside GM1 + TR–DHPE/Bodipy‐Chol (60/10/25/4/1 + 1/1 mol%). Prior to the SMase‐mediated reaction, cholesterol was added to the lipid membranes via methyl‐*β*‐cyclodextrin.

As the *γ* domains shown in Figures 2 and 3 are noncircular, we postulated that they were ceramide‐rich gel domains, which were known to be created in the lipid membranes by SMase‐mediated hydrolysis.^[^ ^33^, ^34^, ^56^, ^57^ ^]^ To confirm this, we investigated the cholesterol content in the *γ* domains, because it is reported that the ceramide‐rich gel domains are not enriched in cholesterol.^[^ ^58^, ^59^, ^60^ ^]^ We examined the partitioning of cholesterol in separate membrane regions using 23‐(dipyrrometheneboron difluoride)‐24‐norcholesterol (Bodipy‐Chol), which is a cholesterol analog that is suitable for quantifying cholesterol in lipid membranes.^[^ ^61^, ^62^ ^]^ As shown in Figure S5a (Supporting Information), Bodipy‐Chol is predominantly present in the cholesterol‐rich L_o_ phase in lipid membranes. Visualization of the lipid membranes undergoing SMase‐mediated hydrolysis shows that the *γ* domains are darker than the L_d_ phase in Bodipy‐Chol fluorescence images, as they are nucleated in the L_d_ phase (blue dotted circle of Figure S5b in the Supporting Information). This indicates that *γ* domains contain less cholesterol than other parts of the membranes. As shown in **Figure** **4**, the *γ* domains continue to contain less cholesterol than other areas, even after they become gray in color in the TR–DHPE fluorescence images. Therefore, the irregular shape and low proportion of cholesterol in *γ* domains confirmed that they were ceramide‐rich gel domains.

Our explanation that the *γ* domains are the ceramide‐rich domains, corresponds to the membrane phase diagram shown in the previous study.^[^ ^63^ ^]^ Because the L_d_ phase is rich in unsaturated phospholipids but lacks SM and cholesterol, it can be simplified to the L_d_ phase in the binary unsaturated phospholipid/SM mixture. Based on this simplification, the L_d_ phase where SMases hydrolyze SMs to ceramides can be described using the phase diagram of the ternary mixture of an unsaturated phospholipid, SM, and ceramide, as shown in Figure S6 (Supporting Information). According to this phase diagram, the SMase reaction is likely to go through a kinetic transition from the single L_d_ phase to the phase‐separated region where the L_d_ phase and the ceramide‐rich gel phase coexist. This indicates that the *γ* domains nucleated in the L_d_ phase are supposed to be the ceramide‐rich gel phase.

The *δ* domain has a higher Bodipy‐Chol intensity than the L_d_ phase, as shown by the red arrows in Figure 4, and appears to be as cholesterol‐rich as the L_o_ phase. However, there are small areas of low Bodipy‐Chol intensity at the L_d_–L_o_ interface and the L_o_–*δ* interface, as indicated by the yellow arrows and the white arrows in Figure 4, respectively, which indicates that they are cholesterol‐poor (i.e., ceramide‐rich). This finding demonstrates that ceramide‐rich domains are formed at such interfaces, which is consistent with previous studies.^[^ ^35^, ^64^, ^65^ ^]^ These ceramide‐rich gel domains are likely to form a rigid wall at the phase boundaries, such that the L_o_ phase and *δ* domain have partially rough interfaces.

Meanwhile, the ceramide‐rich gel domains were not observed within the cholesterol‐rich L_o_ phase (Figure 4), which is in line with the previous studies that examined the effect of cholesterol on the formation of the ceramide‐rich gel phase.^[^ ^36^, ^66^, ^67^, ^68^, ^69^ ^]^ According to the ternary SM/cholesterol/ceramide mixture^[^ ^68^, ^69^ ^]^ and the quaternary 1‐palmitoyl‐2‐oleoyl‐*sn*‐glycero‐3‐phosphocholine (POPC)/SM/cholesterol/ceramide mixture,^[^ ^36^, ^66^, ^67^ ^]^ the ability of ceramides to form the ceramide‐rich gel domains is decreased in the L_o_ phase at higher cholesterol concentrations.These results clearly show that ceramides generated from SMs of the L_o_ phase are likely to dissolve in the cholesterol‐rich L_o_ phase without forming the ceramide‐rich gel domains.

### 2.5 Transverse Organization of Separate Membrane Domains

We deduced the transverse organization of *γ* and *δ* domains by quantifying their diverse fluorescence intensities. We defined the relative fluorescence intensity as *I*_r_  =  (*I*  −  *I*_d_)/(*I*_b_  −  *I*_d_), where *I*, *I*_d_, and *I*_b_ are the fluorescence intensity of the targeted, the darkest, and the brightest domains in the lipid membranes, respectively. *I*_r_ has a value between 0 and 1, and describes how intense the fluorescence of the targeted domain is compared to the brightest and darkest domains. For example, if a targeted domain has a relative TR–DHPE fluorescence intensity ($I_{r}^{\mathrm{TR}}$) of 1, this means that its fluorescence intensity is as high as that of the brightest L_d_ phase, whereas if it has a $I_{r}^{\mathrm{TR}}$ of 0, this means that its fluorescence intensity is as low as that of the darkest L_o_ phase.

**Figure 5:**
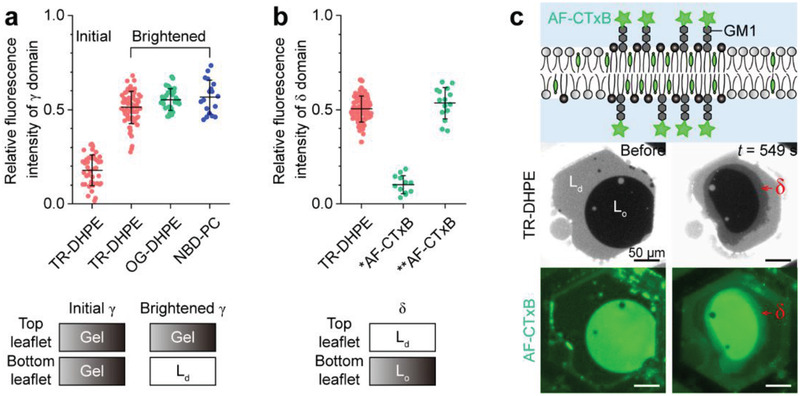
Transverse organization of *γ* and *δ* domains. a) Relative fluorescence intensity of initial and brightened *γ* domains in lipid membranes containing different fluorescently labeled lipids. b) Relative fluorescence intensity of the *δ* domains in lipid membranes labeled with TR–DHPE, lipid membranes in which only the top leaflet is labeled with AF–CTxB (*), and lipid membranes in which both leaflets are labeled with AF–CTxB (**). The values in (a) and (b) are expressed as means ± standard deviations (the number of samples = 18–69 for (a) and 12–112 for (b)). c) Representative fluorescence images of both leaflets of the lipid membranes cross‐linked with AF–CTxB and subjected to SMase‐mediated hydrolysis. The time (*t*) indicates the time elapsed since SMase began to remodel the lipid membranes. The oil used to make the lipid membrane comprised dioleoyl phosphatidylcholine/dioleoyl phosphatidylserine/sphingomyelin/dipalmitoyl phosphatidylserine/ganglioside GM1 + TR–DHPE (55/10/30/4/1 + 1 mol%). Prior to the SMase reaction, cholesterol was added to the lipid membranes via methyl‐*β*‐cyclodextrin.

Thus, we measured the TR–DHPE fluorescence intensity of the *γ* domains, and as shown in **Figure** **5**a, their $I_{r}^{\mathrm{TR}}$ is initially 0.18 ± 0.08, but changes to 0.51 ± 0.08 after they are brightened. This variation in $I_{r}^{\mathrm{TR}}$ indicates that the initial *γ* domains contained as little TR–DHPE as the L_o_ phase, but the brightened *γ* domains contained an amount of TR–DHPE that was intermediate between the amounts contained in the L_d_ and L_o_ phases. This intermediate *I*_r_ value has also been observed in lipid membranes containing other fluorescently labeled lipids, such as Oregon Green 488–1,2‐dihexadecanoyl‐*sn*‐glycero‐3‐phosphoethanolamine (OG–DHPE) and 1‐palmitoyl‐2‐{12‐[(7‐nitro‐2‐1,3‐benzoxadiazol‐4‐yl)amino]dodecanoyl}‐*sn*‐glycero‐3‐phosphocholine (NBD‐PC) ($I_{r}^{\mathrm{OG}}$ = 0.55 ± 0.06, $I_{r}^{\mathrm{NBD}}$ = 0.57 ± 0.09) (Figure 5a), which indicates that this intermediate *I*_r_ value is not due to the physiochemical properties of TR–DHPE. Because fluorescently labeled lipids do not partition into highly ordered phases,^[^ ^32^, ^35^, ^67^, ^70^, ^71^ ^]^ the consistently intermediate *I*_r_ value, independent of the type of fluorescently labeled lipids, implies that the brightened *γ* domains have a transversely asymmetric structure. That is, the top membrane leaflet is the ceramide‐rich gel phase, and the bottom leaflet is the L_d_ phase. This suggests that initially both leaflets of the *γ* domains are gel phases, but the bottom leaflet is subsequently converted into a L_d_ phase as the ceramide concentration increases in the top leaflet via SMase‐mediated hydrolysis.

As illustrated in Figure 5b, the *δ* domains also have an intermediate $I_{r}^{\mathrm{TR}}$ (= 0.50 ± 0.06), which means that they are also transversely asymmetric: one membrane leaflet contains the highly ordered phase, and the other contains the L_d_ phase. As shown in Figure 4, the *δ* domain contains considerably more cholesterol than the L_d_ phase, which indicates that the highly ordered phase is the cholesterol‐rich L_o_ phase. Similarly, the GM1 concentration in the top leaflet of the *δ* domain is almost the same as that in the L_d_ phase (Figure 3b,d), which indicates that the top leaflet of the *δ* domain contains the L_d_ phase. This implies that the bottom leaflet of the *δ* domain contains the L_o_ phase. To further confirm this, we demonstrated the AF–CTxB fluorescence intensity of *δ* domains in lipid membranes in which both leaflets were combined with AF–CTxB (Figure 5c). Before forming the lipid membranes, we preliminarily injected AF–CTxB solution into the aqueous buffer to include AF–CTxB in the aqueous buffer below the lipid membranes, which resulted in the fluorescent labeling of both membrane leaflets with AF–CTxB (Figure S7, Supporting Information). As shown in Figure 5b, the *δ* domain has an AF–CTxB fluorescence intensity that is intermediate between that of the brightest L_o_ phase and the darkest L_d_ phase ($I_{r}^{\mathrm{AF}}$ = 0.53 ± 0.08). These results clearly indicate that the *δ* domain contains the L_d_ phase in its top leaflet and the L_o_ phase in its bottom leaflet.

### 2.6 Overall Membrane Remodeling Processes

The existence of a series of *γ*‐domain formation processes can be deduced from the fluorescence images, the phase information, and the transverse organization of *γ* domains introduced in the previous figures (Figures 3, 4, and 5a), as depicted in Figure S8a (Supporting Information). First, SMases initiate ceramide production in the top membrane leaflet of the L_d_ phase, which results in the aggregation of GM1, SM, and ceramide into the top leaflet of the *γ* domain. As both leaflets of the *γ* domain initially form a gel phase (Figure 5a), a SM/GM1‐rich gel phase is likely to be formed at the bottom leaflet of the *γ* domain via interleaflet coupling. The *γ* domain then grows to a size of several micrometers via the assembly of GM1 and SM. However, as the *γ* domain brightens in TR–DHPE fluorescence images, the bottom leaflet of the *γ* domain gradually changes from a SM/GM1‐rich gel phase into the L_d_ phase. It is unclear why the *γ* domain is coupled with the L_d_ phase rather than the gel or L_o_ phases, but this may be driven by the negative spontaneous curvature of the ceramide‐rich domains. As the SMase‐mediated reaction progresses, the top leaflet of the *γ* domain becomes increasingly enriched with ceramide. Because microdomains that are highly enriched in ceramide spontaneously form a negatively curved gel phase,^[^ ^57^, ^72^ ^]^ it is thermodynamically favorable for the lipids at the opposite bottom leaflet to adopt an arrangement that follows this negative curvature. Therefore, it is likely to be more favorable for the ceramide‐rich domains to transversely couple with the more flexible L_d_ phase rather than the highly ordered phase. Finally, because the brighten *γ* domain has little AF–CTxB fluorescence intensity (Figure 3a), GM1 appears to discharge from the top leaflet of the *γ* domain, as its bottom leaflet undergoes the gel‐to‐L_d_ phase conversion.

With reference to the ceramide‐rich domains formed near the *δ* domain (Figure 4) and the transverse structure of the *δ* domain (Figure 5b,c), the remodeling process of the L_o_ phase can be explained as shown in Figure S8b (Supporting Information). First, SMases generate ceramide‐rich domains at the L_d_–L_o_ interface, as demonstrated in Figure 4. The top leaflet area of the L_o_ phase presumably decreases during SMase‐mediated SM hydrolysis because the *δ* domain has the L_d_ phase as its top leaflet and the L_o_ phase as its bottom leaflet (Figure 5b,c). This reduction in the area of the L_o_ phase is probably due to the release of ceramides from the L_o_ phase. In the beginning of the SMase reaction, ceramides derived from SMs of the L_o_ phase exist as the L_o_ phase with SM and cholesterol by the dissolution of ceramides in the cholesterol‐rich L_o_ phase, as mentioned before. However, once ceramides are oversaturated in the L_o_ phase, they are likely to move from the L_o_ phase to the surrounding L_d_ phase, leading to a decrease in the area of the L_o_ phase. Indeed, it has been reported that there is a solubility limit for ceramides in the L_o_ phase, and excess ceramides can form the ceramide‐rich domains even at a high cholesterol concentration.^[^ ^66^ ^]^ Hence, after the ceramide saturation in the L_o_ phase, ceramides derived from SMs of the L_o_ phase would diffuse from the L_o_ phase to the L_d_ phase, thus reducing the L_o_ phase area and forming the ceramide‐rich domains. This may account for the late‐onset reduction in the area of the L_o_ phase in the top membrane leaflet and the subsequent increase in the area of ceramide‐rich domains at the L_d_–L_o_ interface (Figure 4). The L_o_ phase in the top membrane leaflet appears to undergo a phase transition at the phase interface, and the resultant less ordered liquid phase may become mixed with the existing L_d_ phase. As the area of the L_o_ phase decreases, GM1 molecules are likely to be accumulated in the L_o_ phase of the top membrane leaflet, due to the preference of GM1 for the L_o_ phase. The areal density of the GM1 clustering doubles, and persists at this density. If the L_o_ phase area continues to decrease, the overcrowded GM1 molecules may be released from the L_o_ phase into the L_d_ phase.

## 3 Discussion

It is well recognized that the SMase‐mediated hydrolysis of SM to ceramide in the outer plasma membrane leaflet initiates the clustering of various signaling biomolecules into spatial membrane domains.^[^ ^2^, ^3^, ^10^, ^11^, ^12^, ^13^, ^14^, ^15^, ^16^, ^17^ ^]^ The clustering dynamics of these signaling biomolecules has profound effects on the subsequent transduction of signals into cells and the resulting cellular responses, but how the SMase reaction localizes the signaling biomolecules has been little explored at a fundamental level. Our findings reveal two discrete mechanisms by which SMase triggers the aggregation of GM1 in the heterogeneous top membrane leaflet. In the first mechanism, GM1 clusters into ceramide‐rich domains, which are rapidly nucleated in the cholesterol‐poor L_d_ phase at the beginning of the SMase reaction. These GM1‐rich/ceramide‐rich domains grow to several micrometers in size, but the dense arrangements of GM1 molecules gradually redisperse as the bottom leaflet of the ceramide‐rich domains changes from gel to L_d_ phase. In the second mechanism, GM1, which is predominantly present in the cholesterol‐rich L_o_ phase, becomes condensed, as the area of the L_o_ phase decreases due to a release of ceramides from the L_o_ phase to the L_d_ phase. This second type of GM1 clustering occurs later, but is more effective, as the resulting condensed domains contain much higher concentrations of GM1 and are much longer lasting than those formed by the first mechanism.

From a biological point of view, it is well‐known that plasma membrane components are heterogeneously and dynamically distributed, and are organized into laterally spatial assemblies of cholesterol, SM, and GM1, called lipid rafts.^[^ ^73^, ^74^ ^]^ It is generally accepted that the clustering of signaling molecules in these lipid rafts is due to the SMase‐mediated conversion of the lipid rafts to ceramide‐rich domains, following their coalescence.^[^ ^3^, ^13^, ^21^, ^31^, ^72^, ^75^ ^]^ However, this hypothesis has been controversial due to the lack of direct evidence and several contradictory findings from previous studies. For example, it has been reported that the formation of the ceramide‐rich domains might be inhibited in the cell membrane due to its high cholesterol concentration of 20–50 mol%.^[^ ^36^, ^66^, ^67^, ^76^, ^77^ ^]^ In addition, it has been demonstrated that ceramides could not have the ability to induce the coalescence of the lipid rafts, which raises the possibility that the large clusters of signaling molecules may not be present in the ceramide‐rich domains.^[^ ^67^ ^]^

We believe that this study has biological significance, suggesting the underlying principles of the SMase‐mediated receptor clustering, which have remained unclear to date. Our results indicate that SMase activation can induce the GM1 clustering through distinct mechanisms at different spatiotemporal scales throughout the plasma membrane. First, as outlined in the first mechanism, ceramide‐rich domains are directly derived from nonlipid raft regions considered less ordered phases in the plasma membranes, not from lipid rafts. These ceramide‐rich domains should rapidly aggregate GM1 from a nanosized seed and thus grow to a few micrometers in size, as reported in previous studies.^[^ ^19^, ^74^ ^]^ Second, as outlined in the second mechanism, SMase triggers the reduction in the area of the lipid rafts, thereby condensing GM1 present in the lipid rafts. Since the size of the lipid rafts is extremely smaller (≈20–200 nm)^[^ ^73^, ^74^ ^]^ than the L_o_ phase of our membrane system (O(10 µm)), the GM1 clusters may be present as a nanosized object and thus concentrate faster than the L_o_ phase of our membrane system. This mechanism implies that GM1 can be effectively condensed in lipid rafts without their being transformed into ceramide‐rich domains. Therefore, the GM1 condensation processes induced by both mechanisms might synergistically facilitate signal transmission, because these processes occur at different times and generate different extents of condensation.

Indeed, the SMase‐mediated membrane remodeling and site‐specific, time‐dependent GM1 clustering occur on a comparable timescale (O(1–10 min)) to SMase‐mediated cell signaling, such as apoptosis.^[^ ^78^ ^]^ In the early stage of apoptosis, SMases generate ceramides and induce receptor aggregation in the plasma membranes over a few minutes.^[^ ^3^, ^10^, ^11^ ^]^ As ceramides are generated by SMase‐mediated hydrolysis, they remodel the membrane organization laterally and transversely on a timescale of from a few minutes to tens of minutes.^[^ ^32^, ^64^, ^79^, ^80^ ^]^ This membrane reorganization can induce the formation of the negative spontaneous curvature derived from the ceramide‐rich domains, which may result in membrane blebbing and apoptotic body creation in the late stage of apoptosis.^[^ ^56^, ^57^, ^81^ ^]^ The sophisticated evolution of spatial domains and lipid or protein clusters upon the SMase reaction may serve multiple distinct functions of SMase‐mediated apoptosis at different spatiotemporal scales.

In summary, we have examined the unique ability of ceramide, generated from SM by SMase, to recruit GM1 within the top leaflet of lipid membranes. We used fluorescence microscopy to monitor the dynamic redistribution of GM1 in real time in the heterogeneous top membrane leaflet during SMase‐mediated hydrolysis of SM. This revealed striking phenomena that have not previously been reported, including the formation of two new membrane domains, the antiregistration of the separate membrane regions, and the localization of GM1. By quantifying the diverse fluorescence intensities of the separate phases and domains, we identified two different GM1 clustering processes, which occur in the ceramide‐rich domains formed from the L_d_ phase and in the L_o_ phase that mimics lipid rafts, respectively. This comprises compelling evidence for SMase‐induced multiple localization of signaling molecules at different time and density scales, which is dependent on membrane heterogeneity.

## 4 Experimental Section

### Materials

Dioleoyl phosphatidylcholine, dioleoyl phosphatidylserine, egg SM, dipalmitoyl phosphatidylserine, GM1, NBD‐PC, and Bodipy‐Chol were purchased from Avanti Polar Lipids. TR–DHPE, AF–CTxB, and OG–DHPE were purchased from Invitrogen. Other chemicals, including NaCl, CaCl_2_, MgCl_2_, 4‐(2‐Hydroxyethyl)piperazine‐1‐ethanesulfonic acid (HEPES), hexadecane, silicone oil AR 20, methyl‐*β*‐cyclodextrin, and SMase from *Bacillus cereus* were obtained from Sigma‐Aldrich. All the lipids were dissolved in chloroform or the mixture of chloroform and methanol, and stored in a refrigerator at −20 °C. NaCl, CaCl_2_, MgCl_2_, and HEPES were dissolved in distilled water at concentrations of 100 × 10^−3^, 5 × 10^−3^, 2 × 10^−3^, and 10 × 10^−3^ m, respectively, to prepare an aqueous buffer solution, followed by titration to pH 7.4 with a 0.5 m NaOH solution. The aqueous buffer solution was stored in the refrigerator at 4 °C. AF–CTxB and SMase were dissolved in the aqueous buffer at concentrations of 50 µg mL^−1^ and 5 U mL^−1^, respectively, and they were stored in the refrigerator at 4 °C.

### Lipid Oil Solution

The lipids dissolved in a chloroform‐based solvent were mixed in a 4 mL glass vial according to the molar ratio designed in each experiment, and they were dried with a gentle N_2_ purging. The mixture of hexadecane and silicone oil (1:1 v/v) was injected into the vial to dissolve the lipids at a concentration of 5 × 10^−3^ m. This lipid oil solution was sonicated for 60 min at 50 °C, cooled at room temperature, and used in the experiments within one day.

### Freestanding Planar Lipid Membranes

Tens of freestanding lipid membranes were prepared using an ultrastable freestanding planar lipid membrane array, as described in a previous study^[^ ^46^ ^]^ and Figure S1 (Supporting Information). Briefly, a polyacrylamide hydrogel layer was formed on the bottom of a glass cuvette (700.016‐OG, Hellma), and 3 mL aqueous buffer solution was injected into the cuvette. The 10 µL lipid oil solution was gently dropped on the air–aqueous buffer interface and spread on the aqueous buffer, forming a planar layer. Around 5 min after the lipid oil solution was dropped, a hexagonal TEM grid (G100HEX, Gilder Grids), with a surface treated with a hydrophobic coating, was floated on the layer of the lipid oil solution. The hydrophobic surface of the TEM grid was effectively wetted with the lipid oil solution, and thin oil films were formed in the holes of the TEM grid during the next 5 min. This TEM grid was subsequently submerged within the aqueous buffer and located on the hydrogel surface, leading to the formation of lipid monolayers at the interface between the thin oil films and the aqueous buffer. Over the course of about the next 10 min, the thickness of the oil films was spontaneously reduced enough to make the lipid monolayers adhere to each other, which resulted in the formation of lipid bilayer membranes surrounded by a Plateau–Gibbs border. These lipid membranes were heated at 45 °C for at least 40 min and cooled to 23 °C. Around 1 h after cooling, a cholesterol–methyl‐*β* cyclodextrin solution (5.5 × 10^−3^ m, 1:10 mol%) was injected into the aqueous buffer at a concentration of 50 × 10^−6^ m to increase the cholesterol concentration in the lipid membranes to ≈30 mol%.

### GM1 Fluorescent Labeling and SMase‐Mediated Reaction

The prepared AF–CTxB solution was injected at a concentration of 0.5 µg mL^−1^ into the aqueous solution above the lipid membranes. This solution was incubated for at least 30 min to fully combine AF–CTxB with GM1 present in the top leaflet of the lipid membranes. After that, the prepared SMase solution was injected at a 10 mU mL^−1^ concentration into the aqueous solution above the lipid membranes to trigger SMase‐mediated hydrolysis in the top membrane leaflet.

### Membrane Visualization and Image Analysis

The lipid membranes were visualized through an inverted fluorescence microscope (IX73, Olympus) with an iXon EMCCD camera (X‐6880, Andor Solis). All SMase‐induced membrane remodeling was recorded or captured using the screen recording software, Bandicam. The areas and fluorescence intensities of the separate membrane regions were analyzed using the open‐source program, Fiji.^[^ ^82^ ^]^ The fluorescence intensity of the separate regions was calculated as the difference of the mean gray value between each phase and a background.

### Statistical Analysis

The normalized area and normalized fluorescence intensity values were obtained by the normalization to the initial values before the addition of SMase. The data shown in all the graphs were described as means ± standard deviations of at least three independent experiments. The number of samples for each analysis was introduced in each figure legend.

## Conflict of Interest

The authors declare no conflict of interest.

## Supporting information

Supporting Information

Supplemental Video 1

## Data Availability

The data that support the findings of this study are available from the corresponding author upon reasonable request.

## References

[1] R. Göggel , S. Winoto‐Morbach , G. Vielhaber , Y. Imai , K. Lindner , L. Brade , H. Brade , S. Ehlers , A. S. Slutsky , S. Schütze , E. Gulbins , S. Uhlig , Nat. Med. 2004, 10, 155. 14704790 10.1038/nm977

[2] H. Grassmé , A. Jekle , A. Riehle , H. Schwarz , J. Berger , K. Sandhoff , R. Kolesnick , E. Gulbins , J. Biol. Chem. 2001, 276, 20589. 11279185 10.1074/jbc.M101207200

[3] H. Grassmé , V. Jendrossek , A. Riehle , G. von Kürthy , J. Berger , H. Schwarz , M. Weller , R. Kolesnick , E. Gulbins , Nat. Med. 2003, 9, 322. 12563314 10.1038/nm823

[4] H. Grassmé , A. Riehle , B. Wilker , E. Gulbins , J. Biol. Chem. 2005, 280, 26256. 15888438 10.1074/jbc.M500835200

[5] P. Santana , L. A. Peña , A. Haimovitz‐Friedman , S. Martin , D. Green , M. McLoughlin , C. Cordon‐Cardo , E. H. Schuchman , Z. Fuks , R. Kolesnick , Cell 1996, 86, 189. 8706124 10.1016/s0092-8674(00)80091-4

[6] P. A. Lang , M. Schenck , J. P. Nicolay , J. U. Becker , D. S. Kempe , A. Lupescu , S. Koka , K. Eisele , B. A. Klarl , H. Rübben , K. W. Schmid , K. Mann , S. Hildenbrand , H. Hefter , S. M. Huber , T. Wieder , A. Erhardt , D. Häussinger , E. Gulbins , F. Lang , Nat. Med. 2007, 13, 164. 17259995 10.1038/nm1539

[7] D. M. Carter Ramirez , Z. J. Jakubek , Z. Lu , W. W. Ogilvie , L. J. Johnston , Langmuir 2013, 29, 15907. 24308875 10.1021/la403585v

[8] F. M. Goñi , A. Alonso , Biochim. Biophys. Acta, Biomembr. 2006, 1758, 1902. 10.1016/j.bbamem.2006.09.011 17070498

[9] S.‐W. Yoo , A. Agarwal , M. D. Smith , S. S. Khuder , E. G. Baxi , A. G. Thomas , C. Rojas , M. Moniruzzaman , B. S. Slusher , D. E. Bergles , P. A. Calabresi , N. J. Haughey , Sci. Adv. 2020, 6, eaba5210. 33008902 10.1126/sciadv.aba5210 PMC7852391

[10] H. Grassmé , V. Jendrossek , J. Bock , A. Riehle , E. Gulbins , J. Immunol. 2002, 168, 298. 11751974 10.4049/jimmunol.168.1.298

[11] H. Grassmé , A. Cremesti , R. Kolesnick , E. Gulbins , Oncogene 2003, 22, 5457. 12934106 10.1038/sj.onc.1206540

[12] D. Wheeler , E. Knapp , V. V. R. Bandaru , Y. Wang , D. Knorr , C. Poirier , M. P. Mattson , J. D. Geiger , N. J. Haughey , J. Neurochem. 2009, 109, 1237. 19476542 10.1111/j.1471-4159.2009.06038.x PMC2688711

[13] A. Abu‐Arish , E. Pandžić , D. Kim , H. W. Tseng , P. W. Wiseman , J. W. Hanrahan , J. Gen. Physiol. 2019, 151, 834. 31048413 10.1085/jgp.201812143 PMC6572005

[14] A. Abu‐Arish , E. Pandzic , J. Goepp , E. Matthes , J. W. Hanrahan , P. W. Wiseman , Biophys. J. 2015, 109, 85. 26153705 10.1016/j.bpj.2015.04.042 PMC4572494

[15] W. Zundel , L. M. Swiersz , A. Giaccia , Mol. Cell. Biol. 2000, 20, 1507. 10669728 10.1128/mcb.20.5.1507-1514.2000 PMC85322

[16] E. Posse de Chaves , S. Sipione , FEBS Lett. 2010, 584, 1748. 20006608 10.1016/j.febslet.2009.12.010

[17] S. Chiantia , J. Ries , G. Chwastek , D. Carrer , Z. Li , R. Bittman , P. Schwille , Biochim. Biophys. Acta, Biomembr. 2008, 1778, 1356. 10.1016/j.bbamem.2008.02.008 18346453

[18] I. López‐Montero , F. Monroy , M. Vélez , P. F. Devaux , Biochim. Biophys. Acta, Biomembr. 2010, 1798, 1348. 10.1016/j.bbamem.2009.12.007 20026045

[19] Y. Zhang , X. Li , K. A. Becker , E. Gulbins , Biochim. Biophys. Acta, Biomembr. 2009, 1788, 178. 10.1016/j.bbamem.2008.07.030 18786504

[20] B. M. Castro , M. Prieto , L. C. Silva , Prog. Lipid Res. 2014, 54, 53. 24513486 10.1016/j.plipres.2014.01.004

[21] A. E. Cremesti , F. M. Goni , R. Kolesnick , FEBS Lett. 2002, 531, 47. 12401201 10.1016/s0014-5793(02)03489-0

[22] E. I. Posse de Chaves , Biochim. Biophys. Acta, Biomembr. 2006, 1758, 1995. 10.1016/j.bbamem.2006.09.018 17084809

[23] C. Niaudet , S. Bonnaud , M. Guillonneau , S. Gouard , M.‐H. Gaugler , S. Dutoit , N. Ripoche , N. Dubois , V. Trichet , I. Corre , F. Paris , Cell. Signalling 2017, 33, 10. 28179144 10.1016/j.cellsig.2017.02.001

[24] S. A. F. Morad , M. C. Cabot , Nat. Rev. Cancer 2013, 13, 51. 23235911 10.1038/nrc3398

[25] A. Bai , Y. Guo , Cell Death Dis. 2017, 8, e2963. 28749465 10.1038/cddis.2017.360 PMC5550889

[26] M. Hose , A. Günther , H. Abberger , S. Begum , M. Korencak , K. A. Becker , J. Buer , A. M. Westendorf , W. Hansen , Front. Immunol. 2019, 10, 1225. 31214184 10.3389/fimmu.2019.01225 PMC6554418

[27] A. Jana , E. L. Hogan , K. Pahan , J. Neurol. Sci. 2009, 278, 5. 19147160 10.1016/j.jns.2008.12.010 PMC2660887

[28] G. Wang , E. Bieberich , Adv. Biol. Regul. 2018, 70, 51. 30287225 10.1016/j.jbior.2018.09.013 PMC6251739

[29] E. Gulbins , P. L. Li , Am. J. Physiol.: Regul, Integr. Comp. Physiol. 2006, 290, R11. 16352856 10.1152/ajpregu.00416.2005

[30] D. C. Carrer , E. Kummer , G. Chwastek , S. Chiantia , P. Schwille , Soft Matter 2009, 5, 3279.

[31] C. R. Bollinger , V. Teichgräber , E. Gulbins , Biochim. Biophys. Acta, Mol. Cell Res. 2005, 1746, 284. 10.1016/j.bbamcr.2005.09.001 16226325

[32] S. Z. Ira , D. M. C. Ramirez , S. Vanderlip , W. Ogilvie , Z. J. Jakubek , L. J. Johnston , J. Struct. Biol. 2009, 168, 78. 19348948 10.1016/j.jsb.2009.03.014

[33] Y. Taniguchi , T. Ohba , H. Miyata , K. Ohki , Biochim. Biophys. Acta, Biomembr. 2006, 1758, 145. 10.1016/j.bbamem.2006.02.026 16580624

[34] G. Staneva , A. Momchilova , C. Wolf , P. J. Quinn , K. Koumanov , Biochim. Biophys. Acta, Biomembr. 2009, 1788, 666. 10.1016/j.bbamem.2008.10.026 19059203

[35] Ira , L. J. Johnston , Biochim. Biophys. Acta, Biomembr. 2008, 1778, 185. 10.1016/j.bbamem.2007.09.021 17988649

[36] L. C. Silva , A. H. Futerman , M. Prieto , Biophys. J. 2009, 96, 3210. 19383465 10.1016/j.bpj.2008.12.3923 PMC2718262

[37] B. Boulgaropoulos , H. Amenitsch , P. Laggner , G. Pabst , Biophys. J. 2010, 99, 499. 20643068 10.1016/j.bpj.2010.04.028 PMC2905085

[38] H. Ewers , W. Römer , A. E. Smith , K. Bacia , S. Dmitrieff , W. Chai , R. Mancini , J. Kartenbeck , V. Chambon , L. Berland , A. Oppenheim , G. Schwarzmann , T. Feizi , P. Schwille , P. Sens , A. Helenius , L. Johannes , Nat. Cell Biol. 2010, 12, 11. 20023649 10.1038/ncb1999

[39] A. M. Kabbani , K. Raghunathan , W. I. Lencer , A. K. Kenworthy , C. V. Kelly , Proc. Natl. Acad. Sci. USA 2020, 117, 14978. 32554490 10.1073/pnas.2001119117 PMC7334530

[40] R. W. Ledeen , G. Wu , Trends Biochem. Sci. 2015, 40, 407. 26024958 10.1016/j.tibs.2015.04.005

[41] E. Chiricozzi , G. Lunghi , E. Di Biase , M. Fazzari , S. Sonnino , L. Mauri , Int. J. Mol. Sci. 2020, 21, 868. 10.3390/ijms21030868 PMC7037093 32013258

[42] G. Dalton , S.‐W. An , S. I. Al‐Juboori , N. Nischan , J. Yoon , E. Dobrinskikh , D. W. Hilgemann , J. Xie , K. Luby‐Phelps , J. J. Kohler , L. Birnbaumer , C.‐L. Huang , Proc. Natl. Acad. Sci. USA 2017, 114, 752. 28069944 10.1073/pnas.1620301114 PMC5278494

[43] Y. Itokazu , R. E. Pagano , A. S. Schroeder , S. M. O'Grady , A. H. Limper , D. L. Marks , Am. J. Physiol.: Cell Physiol. 2014, 306, C819. 24500283 10.1152/ajpcell.00168.2013 PMC4010808

[44] P. W. Janes , S. C. Ley , A. I. Magee , J. Cell Biol. 1999, 147, 447. 10525547 10.1083/jcb.147.2.447 PMC2174214

[45] R. M. Larive , L. Baisamy , S. Urbach , P. Coopman , N. Bettache , Biochim. Biophys. Acta, Biomembr. 2010, 1798, 389. 10.1016/j.bbamem.2009.11.016 19962956

[46] H. Lee , Y. Lee , S. S. Oh , S. Q. Choi , Small 2020, 16, 2002541. 10.1002/smll.202002541 32924281

[47] E. A. Merritt , S. Sarfaty , F. Van Den Akker , C. L'Hoir , J. A. Martial , W. G. J. Hol , Protein Sci. 1994, 3, 166. 8003954 10.1002/pro.5560030202 PMC2142786

[48] W. B. Turnbull , B. L. Precious , S. W. Homans , J. Am. Chem. Soc. 2004, 126, 1047. 14746472 10.1021/ja0378207

[49] R. Groza , H. Ewers , Proc. Natl. Acad. Sci. USA 2020, 117, 17467. 32641504 10.1073/pnas.2011359117 PMC7395495

[50] P. L. Yeagle , in The Membranes of Cells, Academic Press, Cambridge 2016, pp. 189–218.

[51] I. P. Sugár , P. L. G. Chong , J. Am. Chem. Soc. 2012, 134, 1164. 22196210 10.1021/ja2092322 PMC4120115

[52] S. L. Veatch , S. L. Keller , Biophys. J. 2003, 85, 3074. 14581208 10.1016/S0006-3495(03)74726-2 PMC1303584

[53] J. D. Perlmutter , J. N. Sachs , J. Am. Chem. Soc. 2011, 133, 6563. 21473645 10.1021/ja106626r

[54] B. Dutagaci , J. Becker‐Baldus , J. D. Faraldo‐Gómez , C. Glaubitz , Biochim. Biophys. Acta, Biomembr. 2014, 1838, 2511. 10.1016/j.bbamem.2014.05.024 PMC4137758 24882733

[55] S. A. Pandit , S. Vasudevan , S. W. Chiu , R. J. Mashl , E. Jakobsson , H. L. Scott , Biophys. J. 2004, 87, 1092. 15298913 10.1529/biophysj.104.041939 PMC1304449

[56] I. López‐Montero , M. Vélez , P. F. Devaux , Biochim. Biophys. Acta, Biomembr. 2007, 1768, 553. 10.1016/j.bbamem.2007.01.001 17292325

[57] J. M. Holopainen , M. I. Angelova , P. K. J. Kinnunen , Biophys. J. 2000, 78, 830. 10653795 10.1016/S0006-3495(00)76640-9 PMC1300685

[58] E. L. Megha , J. Biol. Chem. 2004, 279, 9997. 14699154 10.1074/jbc.M309992200

[59] M. R. Ali , K. H. Cheng , J. Huang , Biochemistry 2006, 45, 12629. 17029417 10.1021/bi060610x

[60] C. Yu , M. Alterman , R. T. Dobrowsky , J. Lipid Res. 2005, 46, 1678. 15863835 10.1194/jlr.M500060-JLR200

[61] Z. Li , E. Mintzer , R. Bittman , J. Org. Chem. 2006, 71, 1718. 16468832 10.1021/jo052029x

[62] S. Park , T. N. Sut , G. J. Ma , A. N. Parikh , N.‐J. Cho , J. Am. Chem. Soc. 2020, 142, 21872. 33345541 10.1021/jacs.0c10674

[63] B. M. Castro , R. F. M. de Almeida , L. C. Silva , A. Fedorov , M. Prieto , Biophys. J. 2007, 93, 1639. 17496019 10.1529/biophysj.107.107714 PMC1948048

[64] Ira , L. J. Johnston , Langmuir 2006, 22, 11284. 17154617 10.1021/la061636s

[65] S. Chiantia , N. Kahya , J. Ries , P. Schwille , Biophys. J. 2006, 90, 4500. 16565041 10.1529/biophysj.106.081026 PMC1471841

[66] B. M. Castro , L. C. Silva , A. Fedorov , R. F. M. de Almeida , M. Prieto , J. Biol. Chem. 2009, 284, 22978. 19520848 10.1074/jbc.M109.026567 PMC2755705

[67] L. C. Silva , R. F. M. de Almeida , B. M. Castro , A. Fedorov , M. Prieto , Biophys. J. 2007, 92, 502. 17056734 10.1529/biophysj.106.091876 PMC1751408

[68] J. V. Busto , J. Sot , J. Requejo‐Isidro , F. M. Goni , A. Alonso , Biophys. J. 2010, 99, 1119. 20712995 10.1016/j.bpj.2010.05.032 PMC2920634

[69] J. V. Busto , A. B. García‐Arribas , J. Sot , A. Torrecillas , J. C. Gómez‐Fernández , F. M. Goñi , A. Alonso , Biophys. J. 2014, 106, 621. 24507602 10.1016/j.bpj.2013.12.021 PMC3944645

[70] K. Yokota , T. Ogino , Chem. Phys. Lipids 2015, 191, 147. 26363303 10.1016/j.chemphyslip.2015.09.001

[71] O. M. Schütte , A. Ries , A. Orth , L. J. Patalag , W. Römer , C. Steinem , D. B. Werz , Chem. Sci. 2014, 5, 3104.

[72] S. Zou , L. J. Johnston , Curr. Opin. Colloid Interface Sci. 2010, 15, 489.

[73] I. Levental , K. R. Levental , F. A. Heberle , Trends Cell Biol. 2020, 30, 341. 32302547 10.1016/j.tcb.2020.01.009 PMC7798360

[74] M. Cebecauer , M. Amaro , P. Jurkiewicz , M. J. Sarmento , R. Šachl , L. Cwiklik , M. Hof , Chem. Rev. 2018, 118, 11259. 30362705 10.1021/acs.chemrev.8b00322

[75] H. Grassmé , J. Riethmüller , E. Gulbins , Prog. Lipid Res. 2007, 46, 161. 17490747 10.1016/j.plipres.2007.03.002

[76] J. B. Massey , Biochim. Biophys. Acta, Biomembr. 2001, 1510, 167. 10.1016/s0005-2736(00)00344-8 11342156

[77] M. Fidorra , L. Duelund , C. Leidy , A. C. Simonsen , L. A. Bagatolli , Biophys. J. 2006, 90, 4437. 16565051 10.1529/biophysj.105.077107 PMC1471871

[78] Y. A. Hannun , Science 1996, 274, 1855. 8943189 10.1126/science.274.5294.1855

[79] J. M. Holopainen , M. Subramanian , P. K. J. Kinnunen , Biochemistry 1998, 37, 17562. 9860872 10.1021/bi980915e

[80] L. Chao , A. P. Gast , T. A. Hatton , K. F. Jensen , Langmuir 2010, 26, 344. 19863058 10.1021/la902084u

[81] A. D. Tepper , P. Ruurs , T. Wiedmer , P. J. Sims , J. Borst , W. J. van Blitterswijk , J. Cell Biol. 2000, 150, 155. 10893264 10.1083/jcb.150.1.155 PMC2185573

[82] J. Schindelin , I. Arganda‐Carreras , E. Frise , V. Kaynig , M. Longair , T. Pietzsch , S. Preibisch , C. Rueden , S. Saalfeld , B. Schmid , J.‐Y. Tinevez , D. J. White , V. Hartenstein , K. Eliceiri , P. Tomancak , A. Cardona , Nat. Methods 2012, 9, 676. 22743772 10.1038/nmeth.2019 PMC3855844

[83] I. Artetxe , C. Sergelius , M. Kurita , S. Yamaguchi , S. Katsumura , J. P. Slotte , T. Maula , Biophys. J. 2013, 104, 604. 23442911 10.1016/j.bpj.2012.12.026 PMC3566447

