# Supplementary material for: Sphingomyelinase‐Mediated Multitimescale Clustering of Ganglioside GM1 in Heterogeneous Lipid Membranes
Source: Adv Sci (Weinh). 2021 Sep 2;8(20):2101766. doi: 10.1002/advs.202101766 (PMC8529493; doi:10.1002/advs.202101766)
Supplement: Supplementary file 1 — Supporting Information [file ADVS-8-2101766-s001.pdf]

WILEY-VCH

© 2021 Wiley-VCH GmbH

ADVANCED SCIENCE
Open Access

Supporting Information

for *Adv. Sci.*, DOI: 10.1002/advs.202101766

Sphingomyelinase-Mediated Multi-Timescale Clustering of Ganglioside GM1 in Heterogeneous Lipid Membranes

*Hyun-Ro Lee and Siyoung Q. Choi**

Supporting Information

Sphingomyelinase-Mediated Multi-Timescale Clustering of Ganglioside GM1 in Heterogeneous Lipid Membranes

*Hyun-Ro Lee and Siyoung Q. Choi**

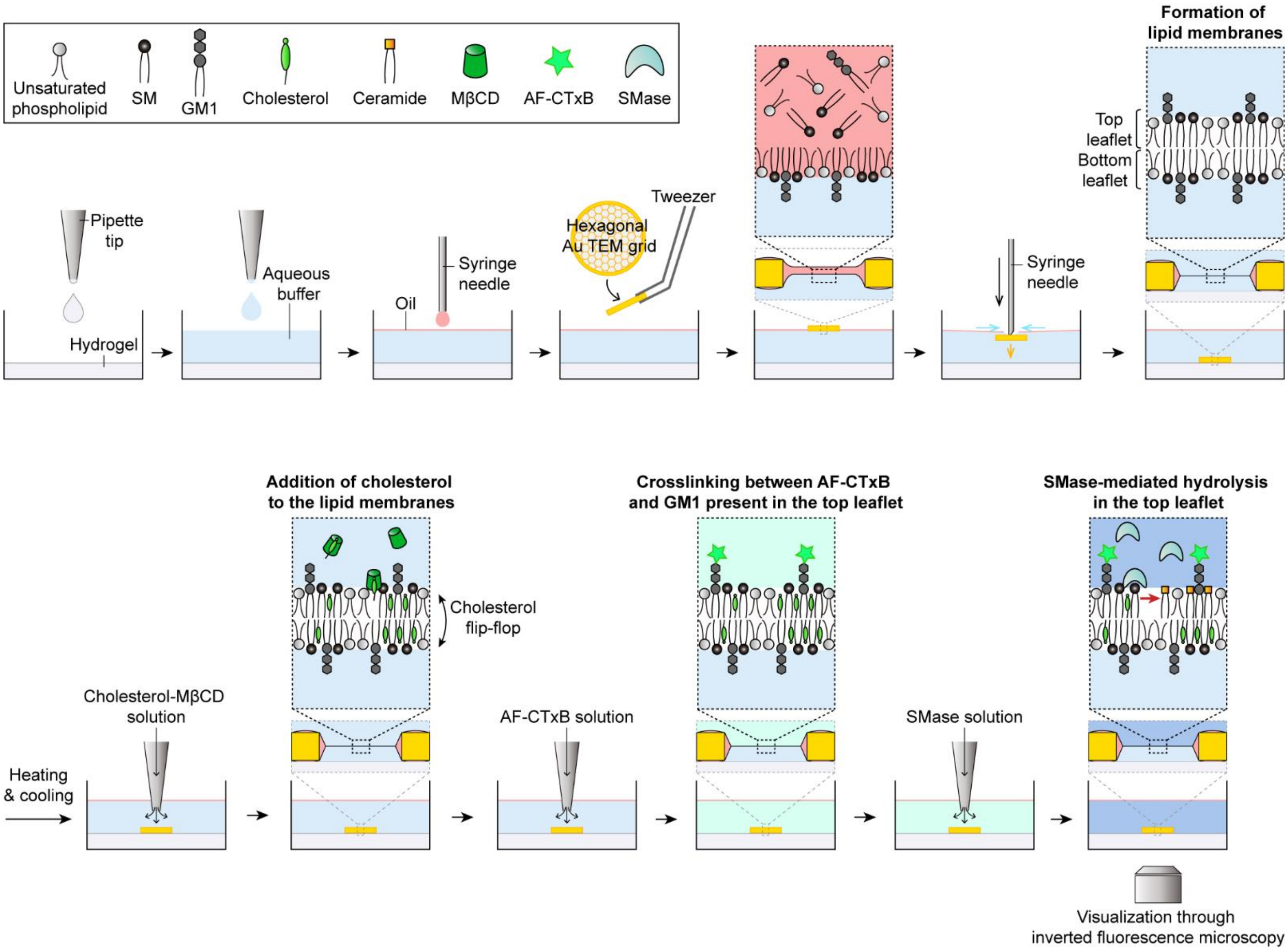

Figure S1. Schematic illustration of our membrane system and experimental procedures. (Where SM = sphingomyelin, GM1 = ganglioside GM1, MβCD = methyl-β-cyclodextrin, AF-CTxB = Alexa Fluor 488-labeled cholera toxin subunit B, SMase = sphingomyelinase, TEM = transmission electron microscopy.)

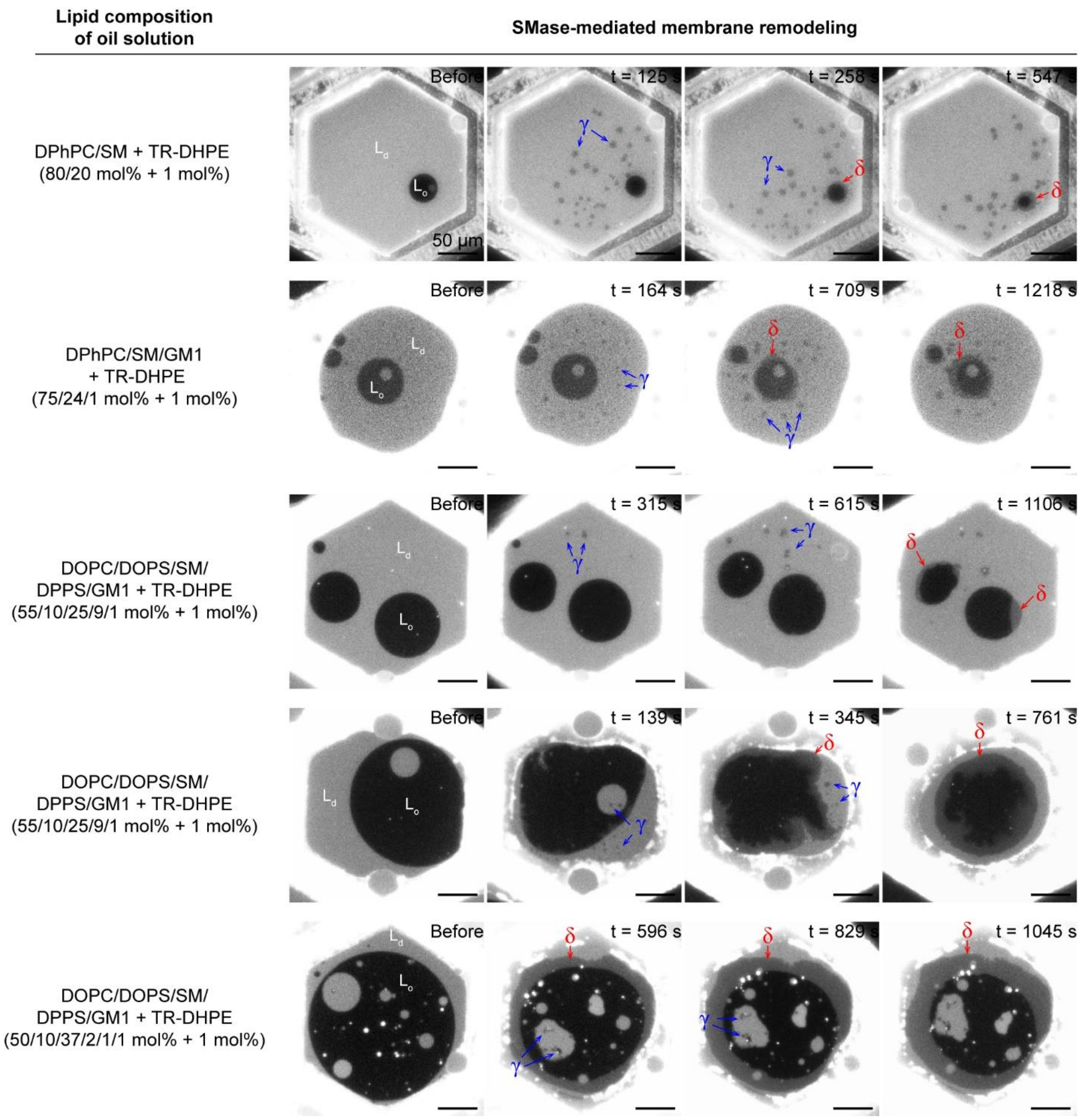

Figure S2. Dependency of sphingomyelinase (SMase)-mediated membrane reorganization on the lipid composition of membranes. Various lipid membranes were created using an oil solution composed of different lipids. The lipid membranes were visualized by Texas Red-1,2-dihexadecanoyl-*sn*-glycero-3-phosphoethanolamine (TR-DHPE) fluorescence. Cholesterol was added to the membranes via methyl-β-cyclodextrin. The time (*t*) displayed on the upper right of each figure indicates the time elapsed since SMase began to remodel the lipid membranes. (Where DPhPC = diphytanoyl phosphatidylcholine, SM = sphingomyelin, GM1 = ganglioside GM1, DOPC = dioleoyl phosphatidylcholine, DOPS = dioleoyl phosphatidylserine, and DPPS = dipalmitoyl phosphatidylserine.)

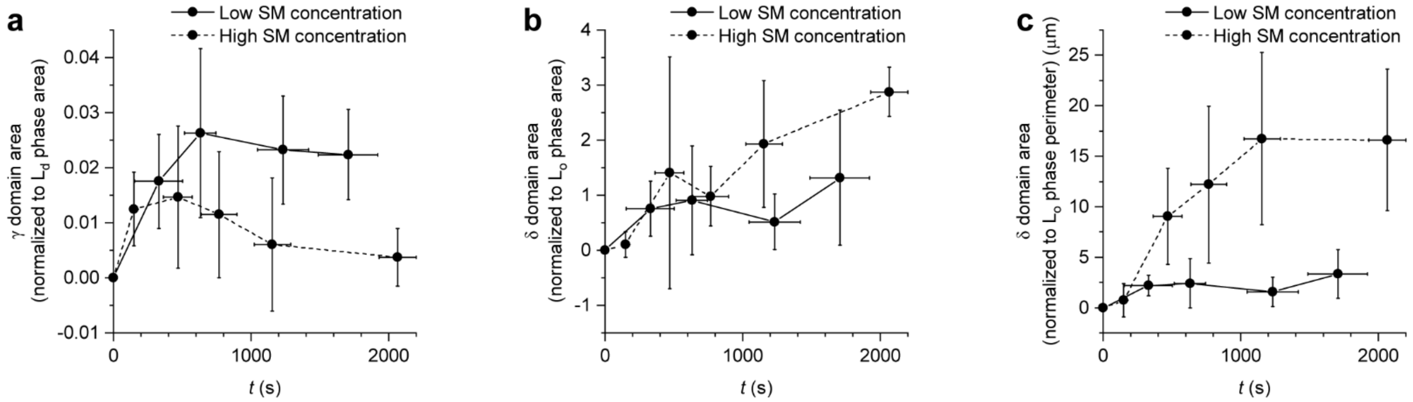

Figure S3. Variation in the normalized areas of the γ and δ domains with time with sphingomyelin (SM) concentration. a) The total area of the γ domains, normalized to the area of the liquid-disordered (L_d) phase. b) The total area of the δ domains, normalized to the area of the liquid-ordered (L_o) phase. c) The total area of the δ domains, normalized to the perimeter of the L_o phase. The *x*-axis of the graphs, *t*, indicates the time elapsed since sphingomyelinase began to remodel the lipid membranes. The values are expressed as means ± standard deviations (the number of samples = 4-7 for (a), 3-7 for (b), and 3-7 for (c)).

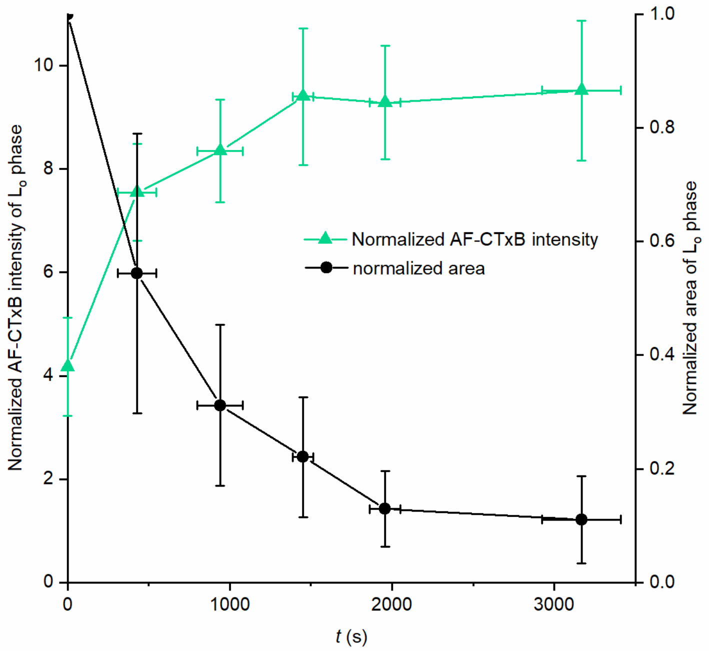

Figure S4. Variation in the Alexa Fluor 488-labeled cholera toxin subunit B (AF-CTxB) fluorescence intensity and normalized area of the liquid-ordered (L_o) phase of the lipid membranes containing high concentrations of sphingomyelin. The intensity plot is taken from Figure 3d. The area values are normalized to the initial L_o phase area. The *x*-axis, *t*, indicates the time elapsed since sphingomyelinase began to remodel the lipid membranes. All of the values are expressed as means ± standard deviations (the number of samples = 4-6). The oil used to form these lipid membranes comprised dioleoyl phosphatidylcholine/dioleoyl phosphatidylserine/sphingomyelin/dipalmitoyl phosphatidylserine/ganglioside GM1 + Texas Red-1,2-dihexadecanoyl-*sn*-glycero-3-phosphoethanolamine (55/10/30/4/1 mol% + 1 mol%).

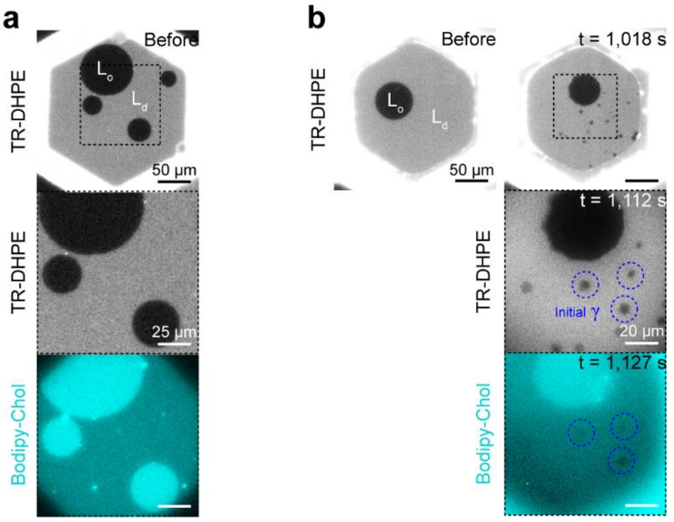

Figure S5. Partitioning of cholesterol in separate membrane regions. The lipid membrane before the sphingomyelinase (SMase) reaction (a) and initial γ domains formed by the SMase reaction (b) were visualized by fluorescent labeling with Texas Red-1,2-dihexadecanoyl-*sn*-glycero-3-phosphoethanolamine (TR-DHPE) and 23-(dipyrrometheneboron difluoride)-24-norcholesterol (Bodipy-Chol). The fluorescence images in the second and third row are expansions of the area in the first row outlined by the black dotted square. The time (*t*) shown in the upper right of each figure indicates the time elapsed since SMase began to remodel the lipid membranes. The oil used to form these lipid membranes comprised dioleoyl phosphatidylcholine/dioleoyl phosphatidylserine/sphingomyelin/dipalmitoyl phosphatidylserine/ganglioside GM1 + TR-DHPE/Bodipy-Chol (60/10/25/4/1 mol% + 1/1 mol% for (a) and 60/10/25/4/1 mol% + 1/1 mol% for (b)).

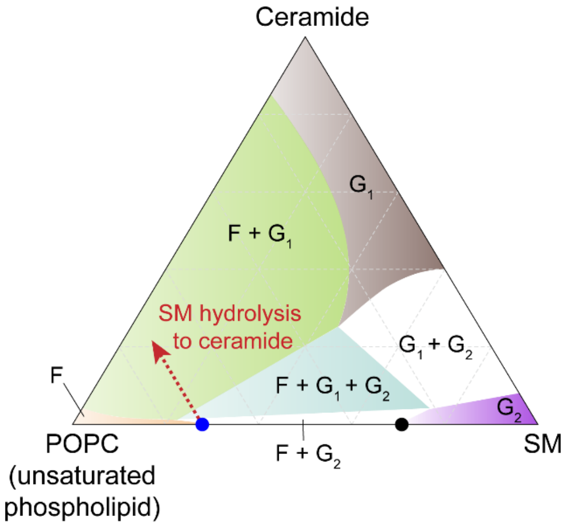

Figure S6. The phase diagram of the ternary mixture of 1-palmitoyl-2-oleoyl-*sn*-glycero-3-phosphocholine (POPC), sphingomyelin (SM), and ceramide. This phase diagram was drawn by referring to the previous study from Castro et al.[63] The abbreviations F, G_1, and G_2 in the phase diagram correspond to the fluid, ceramide-rich gel, and SM-rich gel phases.

Supporting text

As we demonstrated in this study, our lipid membrane system is phase-separated into the L_d and L_o phases. The L_d phase is rich in unsaturated phospholipids but lacks sphingomyelin (SM) and cholesterol; in contrast, the L_o phase is rich in SM and cholesterol but lacks unsaturated phospholipids. Though our membrane system comprises up to six different lipids and 1 mol% of fluorescently labeled lipids, considering the significant components in the L_d phase, the L_d phase can be simplified to the unsaturated phospholipid-rich/SM-poor binary mixture.

Based on this simplification, the L_d phase where SMases generate ceramides can be described using the phase diagram of the ternary mixture composed of 1-palmitoyl-2-oleoyl-*sn*-glycero-3-phosphocholine (POPC), SM, and ceramide (Figure S6).[63] This phase diagram shows that the lipid membrane located in the F + G_2 region is phase-separated into the POPC-rich L_d phase (blue dot) and the SM-rich gel phase (black dot) at the ceramide concentration of 0 mol%. Since POPC is also an unsaturated phospholipid, the L_d phase of our membrane system is supposed to undergo the phase transition similar to the POPC-rich L_d phase. If SMase hydrolyzes one SM molecule to one ceramide molecule within the L_d phase, the location of the L_d phase in the phase diagram is approximately shifted from the blue dot along the dotted red arrow through a kinetic process. Namely, the SMase reaction facilitates the phase transition from the single L_d phase to the phase-separated region where the L_d phase and the ceramide-rich gel phase coexist (F + G_1 region). Therefore, based on this phase diagram, it is concluded that the γ domains nucleated in the L_d phase upon the SMase reaction are supposed to be the ceramide-rich gel phase, consistent with our observation.

It is unknown how exactly the L_d phase and ceramide-rich phase change in composition during the kinetic process, but SMase-generated ceramides are likely to recruit SMs to form the gel domains through the intermolecular hydrogen bonding and chain-chain interaction between ceramide and SM.[67,80,83] When the ceramide concentration increases in

the direction of the dotted red arrow by the sustained SMase reaction (Figure S6), it is anticipated that ceramide concentration in the ceramide-rich gel phase gradually increases in accordance with the phase diagram.

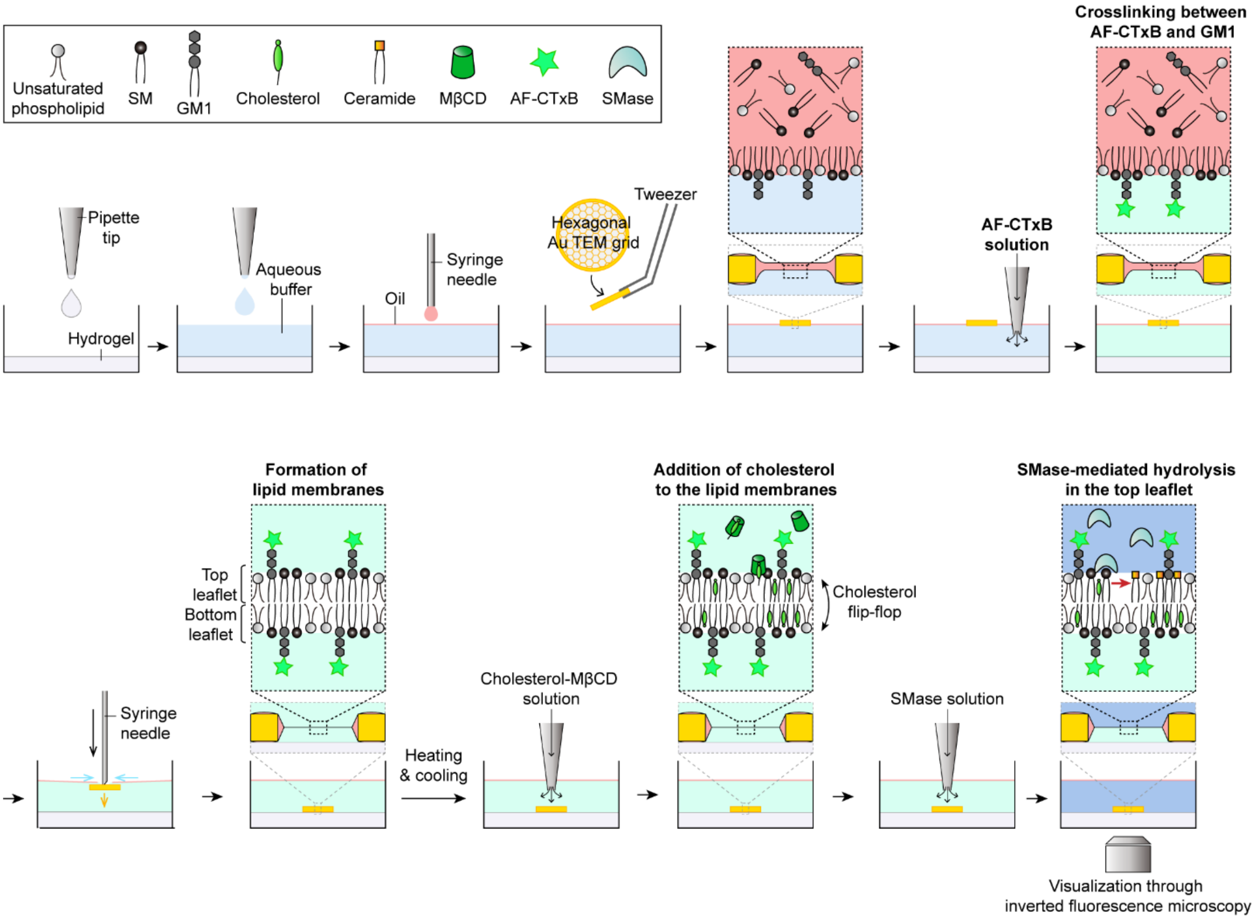

Figure S7. Schematic illustration of our experimental procedures for fluorescent labeling of both membrane leaflets with Alexa Fluor 488-labeled cholera toxin subunit B (AF-CTxB). (Where SM = sphingomyelin, MβCD = methyl-β-cyclodextrin, SMase = sphingomyelinase, TEM = transmission electron microscopy.)

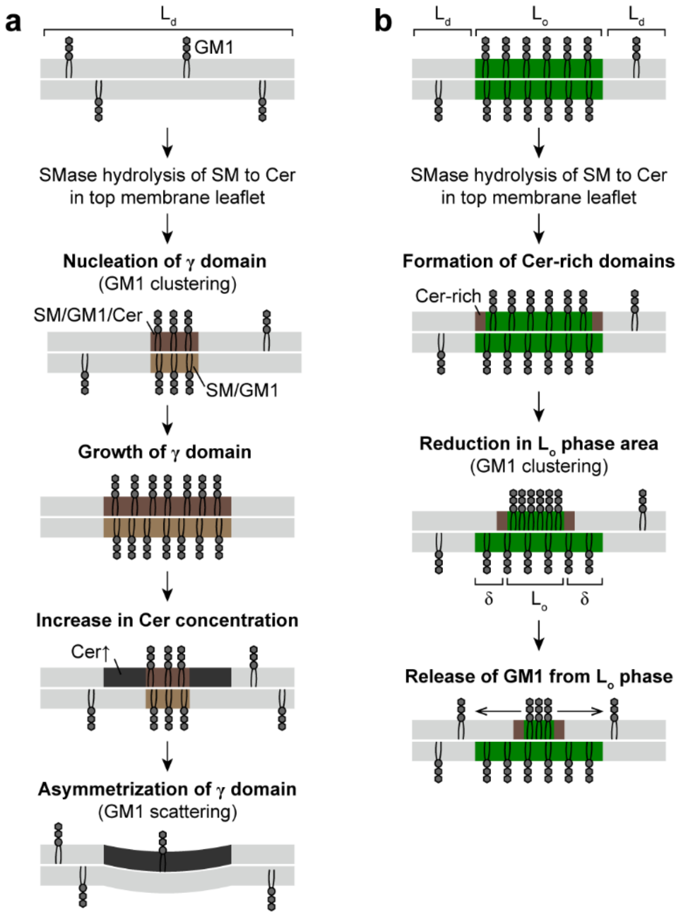

Figure S8. Schematic illustration of the overall process of the GM1 clustering in the γ domain (a) and liquid-ordered (L_o) phase (b). (Where L_d = liquid-disordered; SM = sphingomyelin; Cer = ceramide.)

Video S1. Sphingomyelinase (SMase)-induced remodeling of the lipid membrane shown in Figure 2a. The time (*t*) displayed on the upper right of the screen indicates the time elapsed since SMase began to remodel the lipid membranes. The oil used to form these lipid membranes comprised dioleoyl phosphatidylcholine/dioleoyl phosphatidylserine/ sphingomyelin/dipalmitoyl phosphatidylserine/ganglioside GM1 + Texas Red-1,2-dihexadecanoyl-*sn*-glycero-3-phosphoethanolamine (60/10/25/4/1 mol% + 1 mol%).
